# Implementation of an electronic hospital information system: first results of a longitudinal study

**Published:** 2012-06-15

**Authors:** Rob de Leeuw

**Affiliations:** University Medical Center Utrecht, The Netherlands

**Keywords:** evaluation, implementation, electronic hospital information system

## Abstract

**Introduction:**

June 2011, a new comprehensive Electronic Hospital Information System (CS-EHIS) has been implemented at the University Medical Hospital of Utrecht. The use of this new system may lead to optimal integrated telehealth and telecare; to a new way of working for many employees and to many new challenges in care. Many users have been trained by colleagues (key users). Just before the implementation of CS-EHIS, a baseline measurement has been performed consisting of the completion of a large questionnaire by 100 key users and a small one by a group of about 1000 users. Six months after the baseline measurement a first follow-up questionnaire has been completed by these employees. Users and key users will be asked to complete a questionnaire each six months.

**Aims and objectives:**

The aim of the evaluation of CS-EZIS is to localize and solve possible bottlenecks quickly and to learn from problems examined for future implementations. Main questions of the study are: 1) did the implementation strategy come up to the expectations of professionals, 2) will behavior of professionals and patients change as a consequence of the introduction of CS-EHIS, and 3) will the use of care of patients change as a consequence of the introduction of CS-EHIS.

**Results:**

At the baseline measurement experiences with the old EHIS were examined. The most important complaint of employees was that the system was too slow but overall it was not considered very negatively. The methods of the study, the theoretical model used to study the implementation and preliminary results of the first follow-up measurement will be presented.

**Conclusions:**

A comprehensive evaluation of the implementation of CS-EHIS may lead to a quick localization of bottlenecks.

